# Management of Severe Pediatric Tourette Syndrome Resistant to Drug Treatment

**DOI:** 10.1155/2012/497160

**Published:** 2012-09-23

**Authors:** Hemamali Perera, Chittahari Abayanayaka

**Affiliations:** ^1^University Psychological Medicine Unit, Lady Ridgeway Hospital for Children, Colombo 08, Sri Lanka; ^2^Department of Psychological Medicine, Faculty of Medicine, University of Colombo, Kinsey Road, Colombo 08, Sri Lanka

## Abstract

Tourette syndrome (TS) is a neurodevelopmental disorder with chronic and disabling impacts on multiple domains of functioning in children. Treatment of TS is often complicated by comorbid conditions. We present a 12-year-old boy with severe symptoms of Tourette syndrome (TS), with prominent and markedly disabling vocal tics, who failed to respond to substantial doses of risperidone and haloperidol, given for a prolonged period. Satisfactory outcome was achieved with exposure and response prevention, adjunct to medication. Comorbid ADHD was treated with stimulants with no exacerbation of tics.

## 1. Introduction 

Tourette syndrome (TS) is a neurodevelopmental disorder with a chronic course and is characterized by complex motor and vocal tics [1]. Age of onset of TS is childhood, usually 5 to 10 years of age with a male to female ratio of 5 : 1. Onset of TS is usually with simple motor tics at around 5 years while onset of vocal tics occurs around 8 to 15 years. Tic severity peak in early to mid adolescence and comparatively less in adulthood [2]. Though the main treatment option for TS is pharmacological, management is often complicated by comorbid conditions [3]. 

Accordingly, among children with TS, attention deficit hyperactivity disorder (ADHD) is found in 64%, behavioral problems such as oppositional defiant disorder (ODD) or conduct disorder (CD) in 43%, anxiety problems in 40%, depression 36%, and developmental delay in 28% [4]. Obsessive-compulsive behaviors and obsessive-compulsive disorder (OCD) have been shown to occur among more than one-third of persons with TS [5].

We present a case of an adolescent with severe TS and multiple comorbidities, refractory to pharmacological therapy alone. Our aim is to describe the details of exposure and response prevention (ERP) used in adjunct to medication, to achieve successful symptom control short- and long-term. 

## 2. Case Presentation 

R, a 12-year-old schoolboy and the first born child of nonconsanguineous parents, carried a diagnosis of TS since 8 years of age. He had motor tics affecting the neck, shoulders, and facial grimacing. The most prominent symptom was a very loud bark-like vocalization, which could be heard from some distance away. The vocal tics occurred as frequently as 2-3 times a minute and were highly socially disabling. He presented to our child and adolescent mental health service (outside his area of residence) for the first time at the age of 11 years due to persistence of symptoms despite ongoing treatment. The following details are based on our contact with R. and his parents. 

### 2.1. History

R's symptoms first appeared at the age of 8 years, initially as excessive blinking, which later progressed to jerky repetitive movements involving head and neck regions. The bark-like vocal utterances first appeared 3 years before, which had become louder and more frequent with time. He has had several psychiatric consultations in his local area of residence and had received mainly drug treatment. Information from R's parents and available clinical documentation on previous treatments indicated that R. had been on large doses of medication for many months. These treatments had been initiated with low doses but had been gradually increased due to poor response. Parents reported good compliance with taking medication and close supervision by them. Maximum doses reached were a combination of risperidone 6 mg/day and haloperidol 15 mg/day. These doses provided temporary relief initially but soon ceased to be effective even though they were continued without interruption. R. had missed much of schooling in the previous 3 years because of the vocal utterances, which disturbed everyone around him. The school supported by making special arrangements for lessons but this too could not be sustained. At the time of his presentation to our service (at the age of 12 years and 9 months), R. had not attended school continuously for 3 months, was almost housebound, and avoided social contacts. At the time, R. had taken haloperidol 15 mg/day and risperidone 6 mg/day continuously for over 6 months. 

### 2.2. Assessment

R. was reassessed following admission for inpatient care and the diagnosis of TS was confirmed. In addition to TS, multiple other problems were elicited on clinical and psychometric assessments: (i) prominent symptoms diagnostic of attention deficit hyperactivity disorder (ADHD) combined type, based on DSM IV-TR [1] criteria and SNAP IV scores, (ii) obsessions and compulsions mainly related to cleanliness, method and order, (iii) below average for age in reading, spelling, and verbal expressive language skills (based on standards for Sri Lankan children set by the Institute of Education), and (iv) tendency to overeat with a weight for age was at 85th centile. Obsessions and compulsions did not amount to a diagnosis of obsessive compulsive disorders (OCD). There was no self-injurious behavior and no report of recurrent throat infections. R. has never suffered from any seizure disorders or any other common and recurrent childhood illnesses. Except for a mild delay in beginning to use language, his development has not been of concern to his parents. Because of a positive family history of tics in a second degree male relative on his father's side, R was examined and investigated but considered to be negative for Wilson's disease. A CT brain scan and an EEG did not show any abnormalities. Metabolic screening and liver function tests also were within normal limits.

### 2.3. Pharmacological Therapy

Due to unacceptably high doses of haloperidol taken for many months, the dose was reduced to 6 mg/day. Risperidone was discontinued due to persistence of overeating and to prevent further weight gain. Clonidine 25 *μ*gm/day was added to the drug regime, increased to 50 *μ*gm/day one week later. As the symptoms of ADHD appeared to have a strong negative impact on R's learning, behavior, and the ability to cooperate with planned behaviour therapy, methylphenidate 20 mg/day in 2-divided doses was also added. This had the desired effect of bringing about a noticeable reduction in inattention and impulsiveness. 

### 2.4. Behaviour Therapy

Under inpatient care, therapy was initiated by encouraging R. to identify a premonitory symptom for the vocal tic, which was the most disabling and distressing. Premonitory symptoms are unpleasant sensations of somatosensory nature that prompt a tic. This results in a momentary relief of the sensation. In the first 3 therapy sessions once a day and lasting 30 minutes each, R. was able to identify the premonitory urge as a pricking sensation arising in his epigastric area and reaching up to his throat. R was guided to focus on this premonitory symptom and resist it progressing to a vocal tic by voluntarily suppression. R. adopted a swallowing action at the beginning to help himself to suppress the urge. Starting from an initial 15 minute of suppression of symptom on his own within 5 to 6 days, R. was able go through one full hour without vocalizations by the end of 2 weeks. To keep R. motivated, a reward system contingent on compliance was added. At the time of discharge from hospital after a 4-week stay, R had successfully habituated to the premonitory urge. He had an immediate recurrence of vocal tics in the same intensity as before on attempting to reintegrate back to school. This was overcomed by a one-week break from school, continued practice at home and returning a week later. At one- and 3-month followup, he was having less than 10 vocal tics a day, but low in intensity and not much noticeable in public. At 6 month followup, this too had largely disappeared. R. had by then returned to school full time, was receiving individual support for learning, but was still struggling to cope with academic difficulties, and missed school work. He showed some defiant behaviour with parents at home but not in school. 

## 3. Discussion 

ERP rather than habit reversal was chosen as method of behavioural intervention for several reasons. Firstly, vocal tics were the most prominent and disabling symptom. Secondly, although tics are recognized as involuntary due to its neurodevelopmental basis, they are also described as volitional responses to irresistible and involuntary impulses (premonitory symptoms) [6, 7]. Thirdly, ERP is a useful behavioural technique, known to be as effective as habit reversal in tic disorders, with no rebound phenomena on termination of treatment [8, 9]. Fourthly, R. responded well to this intervention and the improvement was sustained. 

Failure to respond to haloperidol and risperidone in this patient cannot be explained from the available data. Use of clonidine was justified on the basis of the presence of multiple comorbidities and poor response to commonly used drugs. Clonidine improves attention, compulsive behavior, and aggression and is said to benefit 70% of patients with tics [3]. Contrary to previous beliefs, exacerbation of tics with psychostimulants is not an inevitable outcome. Hence, aggressive treatment of comorbid ADHD in TS is recommended [10]. In R., addition of MPH had the desired effect of improving ADHD symptoms with no exacerbation of tics, despite severe symptoms at the beginning. 
